# Valerian and valeric acid inhibit growth of breast cancer cells possibly by mediating epigenetic modifications

**DOI:** 10.1038/s41598-021-81620-x

**Published:** 2021-01-28

**Authors:** Fengqin Shi, Ya Li, Rui Han, Alan Fu, Ronghua Wang, Olivia Nusbaum, Qin Qin, Xinyi Chen, Li Hou, Yong Zhu

**Affiliations:** 1 Department of Environmental Health Sciences, Yale University School of Public Health, New Haven, CT 06520 USA; 2 Department of Oncology and Hematology, Dongzhimen Hospital, Beijing University of Chinese Medicine, Beijing, 100700 China

**Keywords:** Cancer, Drug discovery, Oncology

## Abstract

Valerian root (*Valeriana officinalis*) is a popular and widely available herbal supplement used to treat sleeping disorders and insomnia. The herb’s ability to ameliorate sleep dysfunction may signify an unexplored anti-tumorigenic effect due to the connection between circadian factors and tumorigenesis. Of particular interest are the structural similarities shared between valeric acid, valerian's active chemical ingredient, and certain histone deacteylase (HDAC) inhibitors, which imply that valerian may play a role in epigenetic gene regulation. In this study, we tested the hypothesis that the circadian-related herb valerian can inhibit breast cancer cell growth and explored epigenetic changes associated with valeric acid treatment. Our results showed that aqueous valerian extract reduced growth of breast cancer cells. In addition, treatment of valeric acid was associated with decreased breast cancer cell proliferation, migration, colony formation and 3D formation in vitro in a dose- and time-dependent manner, as well as reduced HDAC activity and a global DNA hypomethylation. Overall, these findings demonstrate that valeric acid can decrease the breast cancer cell proliferation possibly by mediating epigenetic modifications such as the inhibition of histone deacetylases and alterations of DNA methylation. This study highlights a potential utility of valeric acid as a novel HDAC inhibitor and a therapeutic agent in the treatment of breast cancer.

## Introduction

Natural products provide a rich source for the development of novel drugs with anti-tumor activities. Currently, over a half of the available anticancer drugs were derived from botanical sources^1^. For example, Taxol (paclitaxel) is an FDA approved anti-microtubule agent extracted from bark of the Pacific yew tree Taxus brevifolia and has shown a strong anticancer effect in human cancer^2^. Considering herbal extracts and their bioactive compounds are a rich source of therapeutics to human beings, there is a compelling reason to seek novel bioactive compounds in the herbal extracts.

Valerian root is an herb that has long been found to be effective for treating circadian related sleep disorders. The name “Valerian” in Latin means “to be strong or healthy”, which can be attributed to its use as an herbal remedy that has been in practice since ancient Greek and Roman times. Currently, the herb is used mainly as a treatment for insomnia and is considered among the most popular sleeping aids in Europe^3^. Valerian has been approved as a Generally Recognized as Safe (GRAS) food ingredient by the U.S. Food and Drug Administration (FDA). A recent systematic review of research concluded that “The available evidence suggests that valerian might improve sleep quality without producing side effects”^4^.

Disruptions in sleep and circadian rhythm have been shown to be associated with elevated breast cancer risk^5,6^. The International Agency for Research on Cancer (IARC) has designated circadian disruption caused by night shift work as a “2A” carcinogen (probably carcinogenetic) for breast cancer^7,8^. This designation is reaffirmed by the finding of a 40% elevated risk of breast cancer risk among female night shift workers in a recent meta-analysis^6^. In breast tumors, many human circadian genes have been found to be dysregulated^9^. Animal studies have also demonstrated that mice with disruptions in their molecular clocks showed increased breast cancer risk^10^ and accelerated growth rates of malignant tumors^11^.

Given the connection between circadian factors and tumorigenesis, the herb's ability to ameliorate sleep dysfunction may signify a completely unexplored underlying anti-tumorigenic capacity. Valeric acid, a short chain fatty acid (SCFA), is a major active chemical ingredient of valerian and shares a high structural similarity with the known histone deacteylase inhibitor (HDACi) valproic acid^12–14^. HDACi is a novel group of anti-cancer drugs, which may reverse gene-associated DNA methylation and influence gene expression levels^15^. Considering the role of circadian factors in breast cancer tumorogenesis and the potential HDAC inhibiting function, we tested the hypothesis that valeric acid could provide therapeutic benefits against human breast cancer through epigenetic modifications, including DNA methylation and histone deacetylation in the current study.

## Results

### Valerian and valeric acid reduce proliferation of breast cancer cells

**Figure 1:**
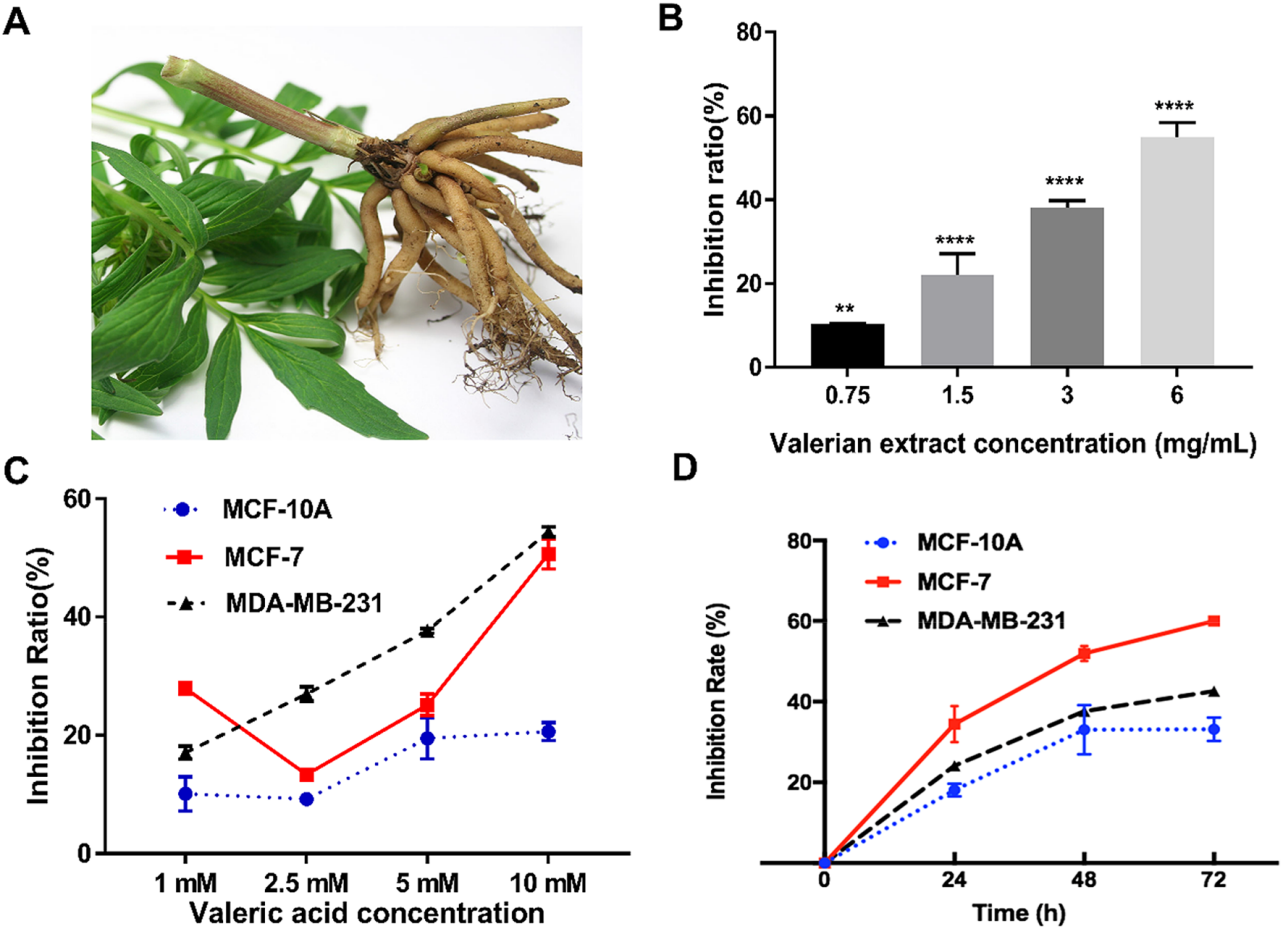
Cell proliferation inhibitory effect of valerian extract and valeric acid on breast cancer cells. (**A**) A picture of Valerian root. (**B**) Valerian inhibited the growth of MCF-7 cells in a dose-dependent manner. MCF-7 cells were incubated with valerian extract in different concentrations (0.75 mg/mL, 1.5 mg/mL, 3 mg/mL, and 6 mg/mL) and significant inhibition of cell proliferation was observed after 48 h. (**C**) Cell proliferation inhibitory effect of valeric acid on MCF-7, MDA-MB-231 and MCF-10A cells at different concentrations (1 mM, 2.5 mM, 5 mM, and 10 mM) and the inhibition of cell proliferation was measured after 48 h. (**D**) Cell proliferation inhibitory effect of valeric acid on MCF-10A, MDA-MB-231 and MCF-7 cells at 24 h, 48 h, and 72 h. Cells were incubated with 5 mM of valeric acid and all experiments were performed in triplicate.

Figure 1A shows a picture of Valerian root. Compared to the control group, cell proliferation rates of valerian treated MCF-7 cells (0.75 mg/mL, 1.5 mg/mL, 3 mg/mL, 6 mg/mL) were significantly inhibited at 48 h by 10.39 ± 0.12% (*p* = 0.0038), 22.16 ± 5.02% (*p* < 0.001), 38.22 ± 1.62% (*p* < 0.001) and 55.01 ± 3.46% (*p* < 0.001), respectively (Fig. 1B). We then tested anti-proliferative activity of valeric acid at different concentrations against breast cancer cell lines MCF-7, MDA-MB-231 and normal breast epithelial cells MCF-10A at 48 h. Results showed that valeric acid (1 mM, 2.5 mM, 5 mM, and 10 mM) inhibited MCF-7 cells by 27.92 ± 1.05% (*p* = 0.009), 13.36 ± 0.60% (*p* = 0.037), 25.15 ± 1.83% (*p* = 0.006), 50.67 ± 2.57% (*p* = 0.001), and inhibited MDA-MB-231 cells by 17.07 ± 1.11% (*p* < 0.001), 26.99 ± 1.21% (*p* < 0.001), 37.65 ± 1.35% (*p* < 0.001), 54.44 ± 0.85% (*p* < 0.001) at 48 h, respectively (Fig. 1C). Valeric acid at 5 mM was further tested for anticancer effect at different time points. Specifically, the proliferation of MCF-7 cells was inhibited by 34.48 ± 3.64% (*p* = 0.007), 51.95 ± 2.04% (*p* < 0.001), and 60.02 ± 0.53% (*p* < 0.001) at 24 h, 48 h, 72 h, respectively, versus 24.13 ± 0.76% (*p* = 0.003), 37.65 ± 0.35% (*p* < 0.001), and 42.16 ± 1.91% (*p* = 0.001) for MDA-MB-231; and 17.01 ± 0.58% (*p* < 0.001), 33.21 ± 3.55% (*p* = 0.016), and 33.18 ± 3.55% (*p* = 0.002) for MCF-10A at the concentration of 5 mM (Fig. 1D). Overall, valeric acid showed strong inhibitory effect on MCF-7 and MDA-MB-231 breast cancer cell lines and relatively less inhibitory effect on the MCF-10A cell line.

### Effect of valeric acid on migration of breast cancer cells

**Figure 2:**
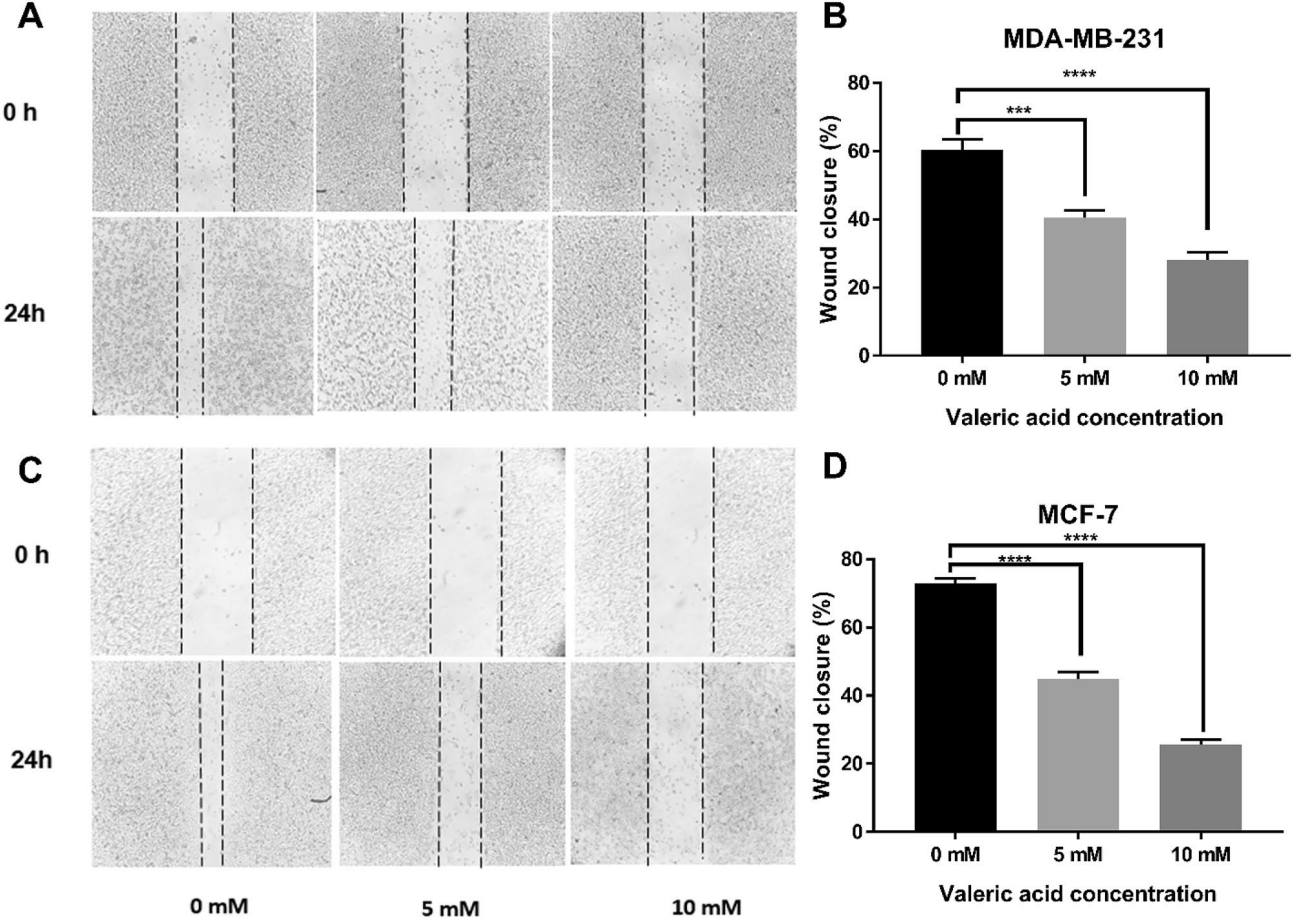
Effect of valeric acid on migration of MCF-7 and MDA-MB-231 cells. Monolayer cells were scraped by a sterile micropipette tip and then treated with various concentrations of valeric acid for 24 h. (**A**,**B**) MDA-MB-231 cells migrated to the wounded region were photographed (100 × magnification). (**C**,**D**) MCF-7 cells migrated to the wounded region were photographed (100 × magnification). The wound closure of the cell cultures was presented in the middle field of each treatment, and data were calculated from three independent experiments. All experiments were performed in triplicate.

A wound healing assay was performed to examine the migration and invasiveness of breast cancer cells treated by valeric acid. For MDA-MB-231 cells, the percentage of wound closure for control, 5 mM and 10 mM groups were 60.48 ± 2.97%, 40.48 ± 2.18% and 28.10 ± 2.18% (Fig. 2A,B), and 72.86 ± 1.43%, 44.76 ± 2.18% and 25.71 ± 1.43% for MCF-7 (Fig. 2C,D), respectively, with a significant difference (*p* < 0.001) in all comparisons.

### Effect of valeric acid on colony formation of breast cancer cells

**Figure 3:**
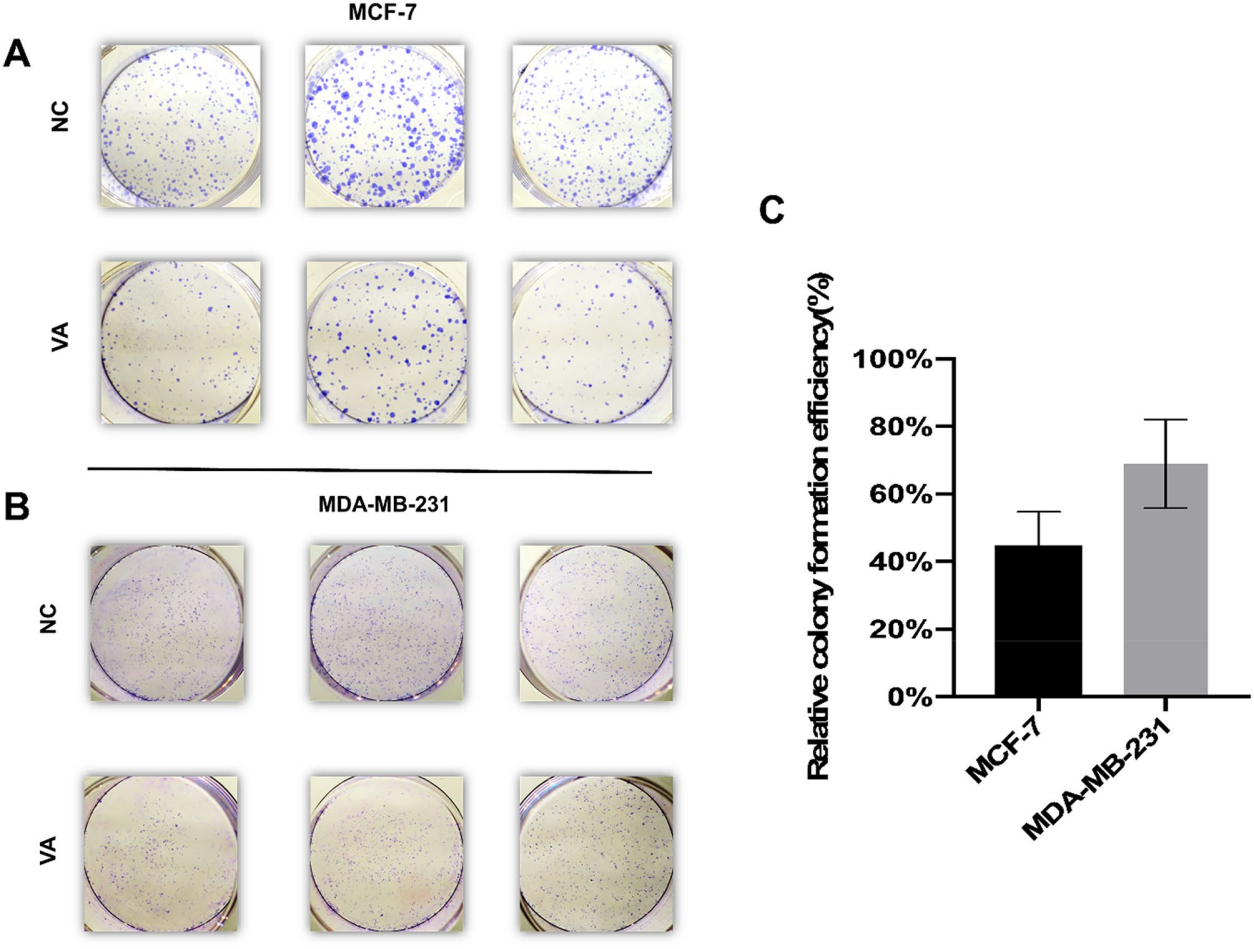
Valeric acid suppresses the colony formation ability of MCF-7 and MDA-MB-231. The representative colony formation of MCF-7 (**A**) and MDA-MB-231 (**B**) cells intervened by valeric acid (VA) and PBS, respectively. The relative colony formation efficiency showed reduction in number of colonies of both cells (**C**). Data are presented as the mean ± standard deviation (SD).

The colony formation assay showed reduced cell colony formation ability of the cells (MCF-7 and MDA-MB-231) in valeric acid treated groups (Fig. 3A,B). Figure 3C revealed that the calculated relative colony formation efficiency was 44.84 ± 8.05% for MCF-7 cells treated by valeric acid (*p* < 0.001) and 68.9 ± 10.72% for MDA-MB-231 cells treated by valeric acid (*p* < 0.001).

### Inhibition of 3D formation by valeric acid

**Figure 4:**
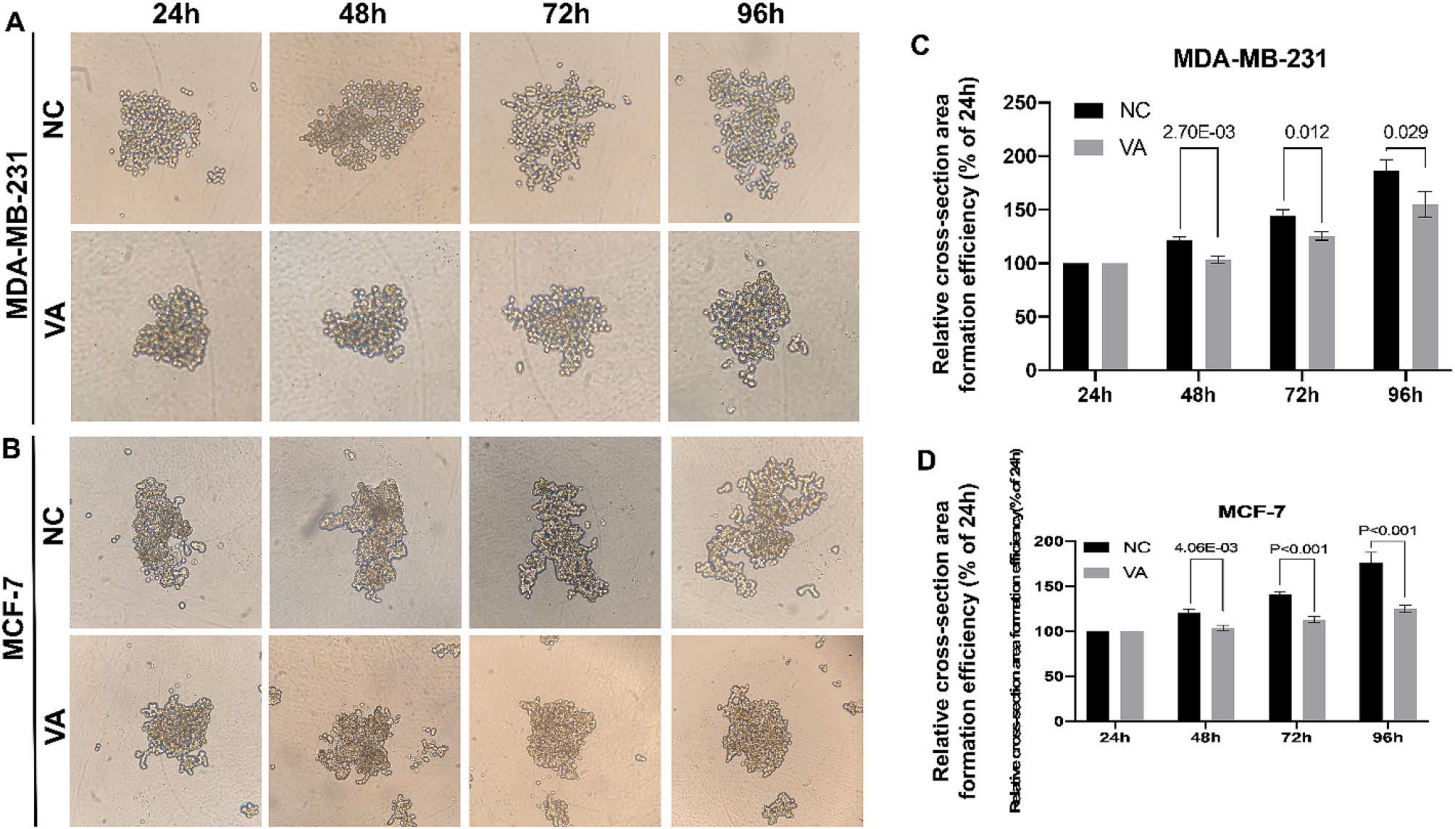
Valeric acid inhibits the 3D formation of MCF-7 and MDA-MB-231 cells. (**A**) illustrated the dynamic changes of 3D formation for MDA-MB-231 cells. (**B**) showed the dynamic changes of 3D formation for MCF-7 cells. Their relative cross-section area formation efficiencies were shown in (**C**,**D**). Significant reduction of cross-section area of MDA-MB-231 cells (**C**) and MCF-7 cells (**D**) in VA treated groups were observed.

To investigate the impact of valeric acid on cell 3D condition that mimics breast cancer cell growth in vivo, the relative cross-section area formation efficiency was calculated by the comparison to their original area size (24 h) in each group, respectively. As shown in Fig. 4, for MDA-MB-231 cells, relative cell cross-section area of valeric acid treated group reached to 103.52 ± 2.77% at 48 h, and showed a significant difference compared to its counterparts of the control group (121.34 ± 2.61%, *p* < 0.001). At 96 h, the trend still remained, relative area of valeric acid treated group reached to 168.54 ± 9.67% compared to 180.73 ± 8.86% of the control group (*p* = 0.029). Similarly, a significantly smaller spheroid area was observed in the valeric acid treated group compared to the control at both 48 h and 96 h (*p* < 0.001) in experiment using MCF-7 cells.

### Effect of valeric acid on HDAC activity

**Figure 5:**
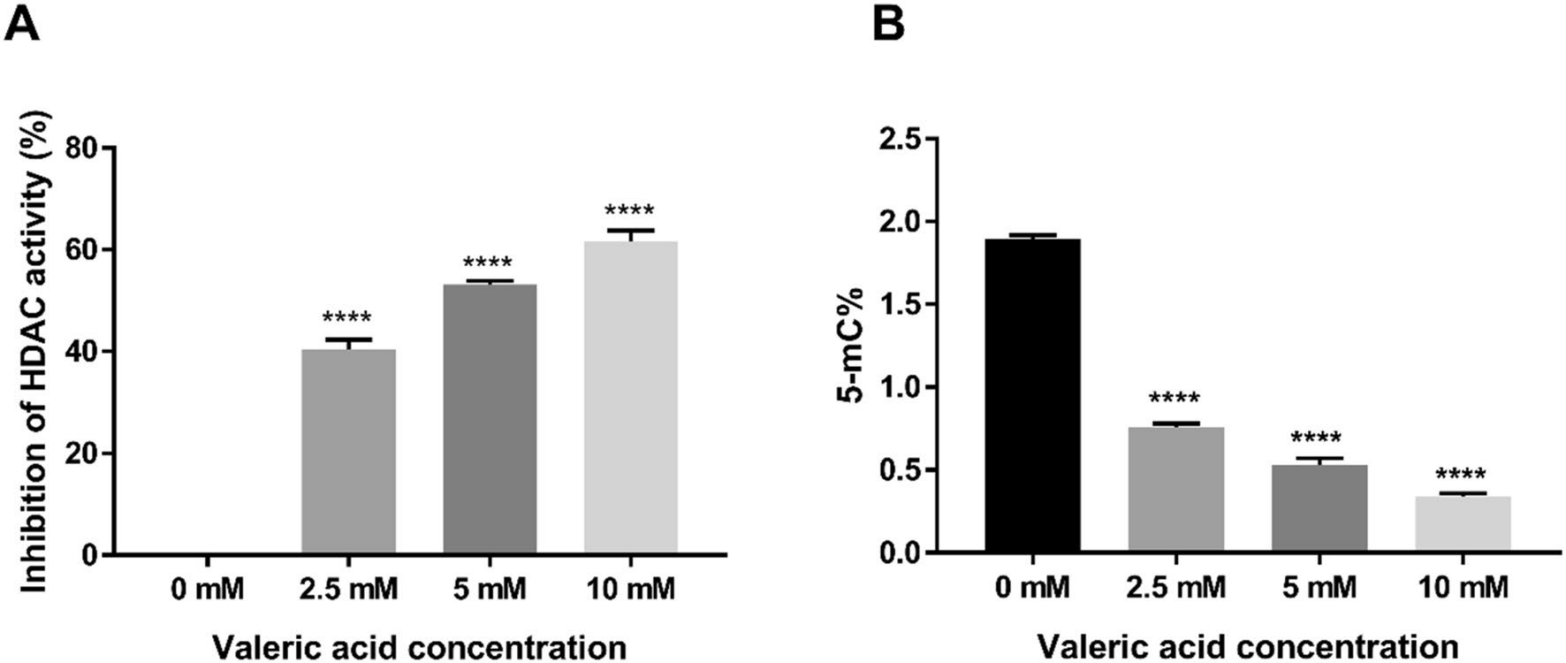
Impact of valeric acid on HDAC activity and global DNA methylation. (**A**) MCF-7 cells were incubated at three different concentrations of valeric acid and HDAC activity was measured after 48 h. Valeric acid significantly inhibited HDAC activity at concentration of 2.5 mM, 5 mM, and 10 mM compared to the control group (*p < 0.05). (**B**) The Global DNA methylation assay revealed that valeric acid (2.5 mM, 5 mM, and 10 mM) significantly decreased the levels of global DNA methylation in MCF-7 cells compared to the control group (*p < 0.05). All assays were performed in triplicate.

MCF-7 cells were treated with 2.5 mM, 5 mM, and 10 mM of valeric acid and HDAC activity was measured after 48 h (Fig. 5A). The HDAC activity inhibition ratio of cells treated with 2.5 mM valeric acid was 40.44 ± 1.88% that showed a significant reduction compared to the control group (*p* = 0.028). Similarly, the HDAC activity of MCF-7 cells was inhibited by 53.15 ± 0.68% (*p* = 0.002) at the concentration of 5 mM valeric acid and 61.64 ± 2.10% (*p* < 0.001) at the concentration of 10 mM valeric acid compared to the control group.

### Effect of valeric acid on global DNA methylation

A dose-dependent effect of valeric acid on the global DNA methylation levels in MCF-7 cells was observed. MCF-7 breast cancer cells were incubated with 2.5 mM, 5 mM, and 10 mM of valeric acid and global DNA methylation was measured at 72 h. Global DNA methylation levels associated with different concentrations of valeric acid were 0.76 ± 0.02% (*p* < 0.001), 0.53 ± 0.04% (*p* < 0.001) and 0.34% ± 0.02 (*p* < 0.001), respectively, versus 1.89 ± 0.03% for the control group in MCF-7 cells (Fig. 5B), indicating decreased global DNA methylation levels associated with increased valeric acid concentrations.

### Epigenetic landscape changes affected by valeric acid

To better understand epigenetic landscape changes affected by valeric acid, we performed the genome-wide DNA methylation assay followed by network-based analysis. Our data showed that 1398 CpG sites were differentially methylated across 1075 unique genes (Supplementary table). Significant CpG sites with methylation differences were determined by adjusted *p* values (FDR *p* < 0.05). Delta-beta ranges from − 0.14 to − 0.52 for hypomethylated sites and from 0.14 to 0.83 for hypermethylated sites. Of these sites, 1267 CpG sites were hypermethylated while 131 CpG sites were hypomethylated in treated cells. According to the reference location of the probes used for each CpG site, we found that 229 CpG sites with differential methylation are located in either 5′UTR or 3′UTR regulatory regions. Sixty-three genes have multiple CpG sites with differential methylation and these CpG sites in 57 of these genes are either all hyper- or hypo-methylated.

**Figure 6:**
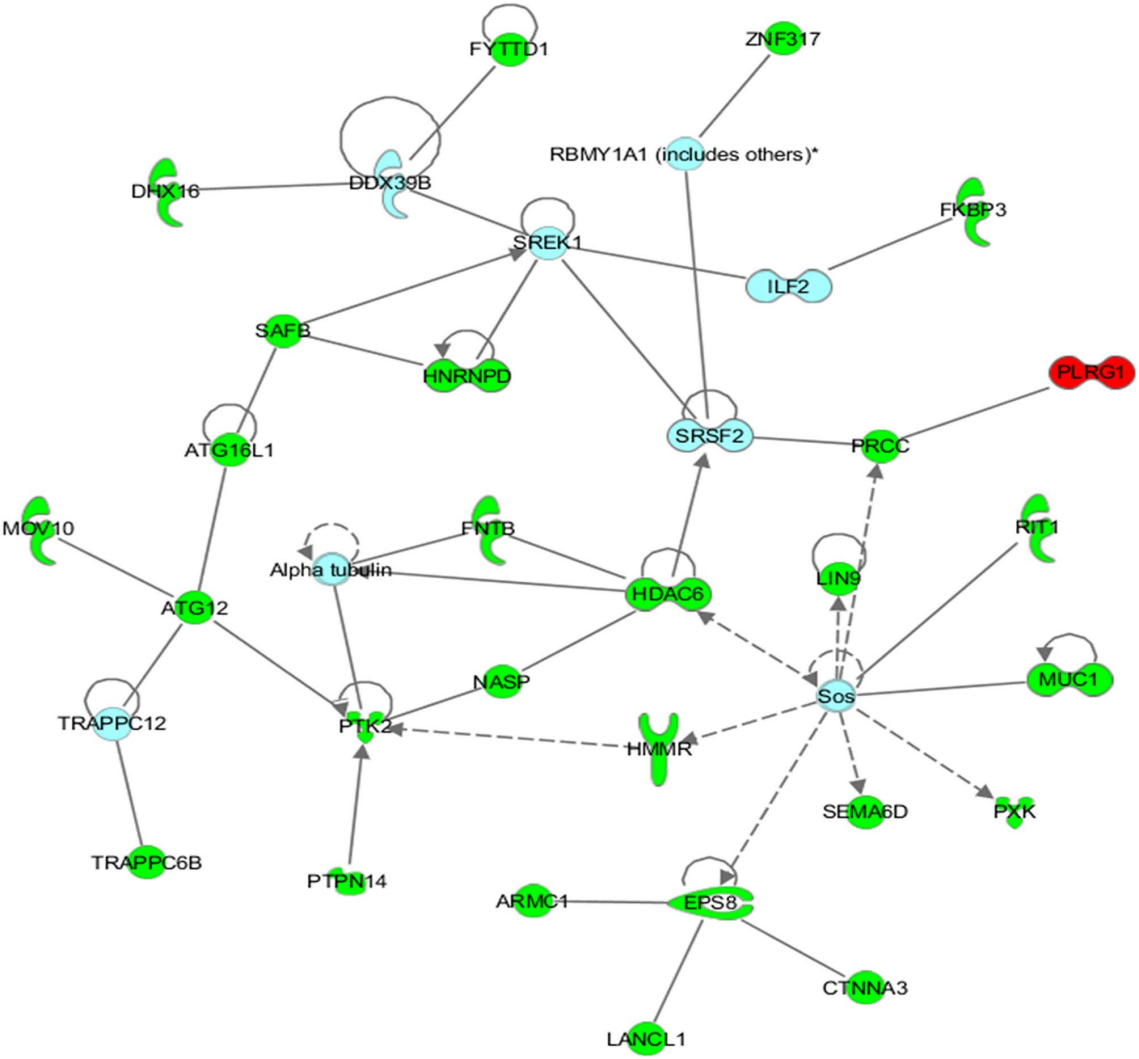
Network of interactions significantly enriched for genes displaying altered methylation patterns following treatment with valeric acid. This network was identified by the IPA as being the most significantly associated with the set of input genes and molecules within this network have functional relevance in “RNA post-transcriptional modification, Cancer, and Developmental disorder” (*p* = 10E−59). Of the 27 genes present in the network, 26 were significantly hypermethylated are shaded in green, while only 1 was hypomethylated are shaded in red. Each interaction is supported by at least one literature reference identified in the Ingenuity Pathway Knowledge Base, with solid lines representing direct interactions, and dashed lines representing indirect interactions.

Among differentially methylated genes, there were 15 histone-related genes including histone deacetylase 6 (HDAC6) that was hypermethlated. The network identified by the IPA tool as being most significantly associated with the set of input genes was designated as having functional relevance in “RNA post-transcriptional modification, cancer, and developmental disorder” (*p* = 10^–59^) as illustrated in Fig. 6. Briefly, 27 genes were present in the network, 26 of which were significantly hypermethylated, while 1 of which was hypomethylated in treated cells. Several putative tumor related genes with oncogeneic role in breast cancer were hypermethylated, such as *HDAC6, NASP, HNRNPC* and *LIN9*.

## Discussion

Despite being the most commonly used herb (or herbal remedy) for sleep disorders, the efficacy of valerian root to treat cancer remained unexplored. We have shown that valerian root and valeric acid (a major compound of valerian root) can inhibit proliferation, migration, colony formation and 3D formation of breast cancer cells in a dose- and time-dependent manner. Moreover, this inhibitory effect was significantly decreased on normal breast epithelial cells compared to breast cancer cells, indicating a cancer-specific action with low side-effects. These findings support our hypothesis that the sleep-related herb, valerian root, may provide therapeutic benefits against human breast cancer.

To explore the underlying basis of the functional effect of valeric acid, we also conducted the HDAC activity assay because valeric acid shares a high structural similarity with the known histone deacteylase inhibitor (HDACi) valproic acid^12–14^. Histone acetylation is an important epigenetic modification and HDAC inhibitors are among the most promising targets in the development of drugs for cancer therapy^16^. More than 15 HDAC inhibitors have been tested in preclinical and clinical studies. Inhibition of HDAC leads to the acetylation of non-histone proteins such as tumor suppressor p53^17^, which may exhibit antiproliferative properties^18,19^. Our data demonstrate that valeric acid significantly decreases HDAC activity in treated breast cancer cells, which provides a possible mechanism to account for its anticancer efficacy. This result suggests that valeric acid may serve as a novel HDACi for breast cancer chemotherapy.

Histone acetylation may modify chromatin and most DNA methylation related proteins (DNA methyltransferase Dnmt1, several methyl-CpG binding proteins, MeCP2, MBD2, and MBD3) are associated with histone deacetylase^20^. These enzymes regulate the process of DNA methylation^15^ and the HDAC activity reduced by valeric acid treatment may lead to alterations of DNA methylation. Our results support this assertion by showing that both global DNA methylation level and gene specific DNA methylation status were affected by valeric acid. Of particlar interest, genome-wide DNA methylation screening detected 15 histone-related genes, including histone deacetylase 6 (HDAC6), to be hypermethylated, which is in congruent with the observed decreased HDAC activity in valeric acid treated cancer cells.

Hypermethylated HDAC6 gene may have anticancer properties because HDAC6 was significantly overexpressed in, not only breast cancer^21^, but also various other tumor types^22^. Histone deacetylase 6 (HDAC6) is a unique member of the HDAC family and plays an important role in regulating the function of HSP90, α-tubulin, and the aggresome^23^ and effects on functional reduction of these downstream oncogenic targets may account for the growth inhibitory effects of HDAC6 knockdown.

We also searched for transcription factor binding sites in the scouse sequence (50 bases) of HDAC6 5′UTR that harbors the hypermethylated CpG site using the PROMO tool^24^ and identified binding sites of 4 transcription factors, Pax-5 (GGGCCTG/CCTGCCC), p53 (CCTGCCC), AP-2 alphaA (GCCTGC), and C/EBP beta (GCAA). All of these 4 transcription factors have been shown to play roles in breast tumorigenesis^25–28^, which warrants further investigation on molecular mechanisms of valeric acid efficacy.

In addition to HDAC6, several other putative tumor related genes with oncogeneic role in breast cancer were hypermethylated after valeric acid treatment in our study. NASP, nuclear autoantigenic sperm protein, has become an important constituent of “poor prognosis signature” in breast cancer patients^29^. Depletion of the histone chaperone tNASP inhibits proliferation and induces apoptosis in prostate cancer PC-3 cells^30^ and human gastric cancer^31^. HNRNPC, heterogeneous nuclear ribonucleoproteins C, is a RNA binding protein and its elevated expression has been reported in cancer cells^32^, but the function of HNRNPC in tumors is still not clear. Repression of HNRNPC in breast cancer cells MCF7 and T47D was reported to inhibit cell proliferation and tumor growth^33^. LIN9 is a part of the MuvB core complex (composed of LIN9, LIN52, LIN37, LIN54, and RBBP4), which functions as a transcriptional regulator of mitosis. Overexpression of LIN9 has been observed in the majority of triple-negative breast cancers (TNBCs) and is associated with poor prognosis across breast cancer subtypes^34^. Hypermethylation of these putative oncogenes may provide a new mechanism accounting for the observed anticancer efficacy of valeric acid in addition to the HDAC mediated processes.

Changes or alterations in both histone acetylation and DNA methylation are common targets studied in cancer epigenomics because these epigenetic processes are generally involved in cell proliferation, differentiation, and survival, and are linked to carcinoma progression^35,36^. Discovering chemicals that can modify these epigenetic processes is a promising new strategy for drug development in cancer chemotherapy. The functional effects of valeric acid in the inhibition of histone deacetylase and alteration of DNA methylation make it a promising novel epigenetic agent for future clinical implications in breast cancer treatment.

Although a profound anticancer effect is observed, findings from this study are still preliminary, which need to be further confirmed in more breast cancer cell lines and animal models. More mechanistic studies are also necessary to further evaluate other epigenetic effects of valeric acid. Inhibition rates of valeric acid on MCF-7 cells (not MDA-MB-231) decrease at low concentrations and then increase again at high doses in cell proliferation assay. This might be due to hormone receptor status of these 2 cells. MCF-7 is a breast cancer cell line with estrogen, progesterone and glucocorticoid receptors and MDA-MB-231 is a triple negative breast cancer cell line. Agents such as valeric acid may have different types of dose-responses (i.e. bell-shaped and U-shaped dose–response curves) when used to treat different tumor types. More breast cancer cell lines and doses are needed to confirm this observation in future study. Nonetheless, as the promising profile of valeric acid were well established, valeric acid is a potential new therapeutic agent for breast cancer and probably cancer in general, worthy of further investigation.

## Materials and methods

### Cell lines and cell culture

Human breast cancer cells MCF-7 and MDA-MB-231, and normal breast epithelial cells MCF-10A were purchased from the American Type Culture Collection (ATCC) (Manassas, VA, USA). MCF-10A cells were maintained in MEGM (Life Technologies, CA, USA) supplemented with 100 ng/mL cholera toxin. MCF-7 and MDA-MB-231 cells were cultured in DMEM (Life Technologies, CA, USA) supplemented with 10% fetal bovine serum, 1% penicillin–streptomycin. The cells were cultured at 37 °C in a humidified atmosphere consisting of 5% CO_2_. The culture medium was changed once every 2 days.

### Preparation of valerian extract and valeric acid

Valerian and valeric acid were purchased from Chromadex (Irvine, CA, USA). Valerian was received as a powder from the herb pulverized rhizome. To prepare the extract of valerian for experiments, 250 mg of this powder was diluted in 2 mL of distilled water, heated to 100 °C for 20 min in a water bath and centrifuged at 1000*g* for 10 min. The supernatant was filtered through a hydrophilic membrane containing 0.22 μM pores (Millipore, Billerica, MA, USA). Valeric acid was diluted in culture medium to obtain working concentrations.

### Cell proliferation assay

We first tested the anticancer effect of valerian on the growth of breast cancer cells using cell proliferation assay. MCF-7 cells were harvested and seeded into each well of 96-well plates at the concentration of 4000 cells/100 μL, and different concentrations of valerian extract (0.75 mg/mL, 1.5 mg/mL, 3 mg/mL, and 6 mg/mL, final concentrations after dilution with medium) or the same volume of ddH_2_O (control group) were added to each well. After incubation for 48 h, we added 20 μL of MTS assay (Promega, Wisconsin, USA) per well in a dark hood and then incubated plates for 2 h. Optical density (OD) was determined at the wavelength of 490 nm by a microplate Spectrophotometer (Biotek, Winooski, USA). MCF-7, MDA-MB-231 and MCF-10A cell lines were used to detect the efficacy of valeric acid on the proliferation of breast cancer cells. Cells were seeded into 96-well plates as described above. 1 mM, 2.5 mM, 5 mM, and 10 mM of valeric acid or the same volume of ddH_2_O were added and OD values were measured at different time points. Triplicate were conducted for each condition at each time point. The proliferation inhibition rate was calculated using the formula as: inhibition ratio = (1 − OD_treated group_/OD_control group_) × 100%. The inhibition ratio was fitted to a non-linear regression plot to determine the IC_50_ (half maximal inhibitory concentration) for breast cancer cells treated with valerian extract or valeric acid.

### Wound-healing assay

In order to understand how valeric acid affects cancer cell migration and confirm the cell proliferation results, we performed a wound-healing assay. Approximately 1 × 10^6^ MCF-7 or MDA-MB-231 cells were added into each well of 6-well tissue culture plates with culture medium containing 10% FBS and grew to nearly 80–90% confluence. A scratch was gently made using a 100 μL pipette tip in each well and the cells were washed with PBS twice to remove the detached cells. Cells were then treated with different concentrations of valeric acid or the same amount of ddH_2_O with 0.1% FBS at 37 °C for 24 h. Wound-healing assay was conducted in triplicate. Images were taken at 0 h and 24 h and the area of a wound was measured with ImageJ software (version of 1.52a) and the average extent of wound closure was quantified.

### Colony formation assay

To examine relatively long-time (~ 10 days) inhibitory impact of valaric acid on the growth of breast cancer calle, we conducted a colony formation assay. Approximately 3000 MCF-7 or MDA-MB-231 cells were seeded into 6-well tissue culture plates with 2 mL complete medium per well. Cells were treated with 0.85 mM valeric acid or the same amount of ddH_2_O (control group). Cells were cultured for 10 days and drug-containing culture medium was renewed once every 3 days. Cells were then fixed by 4% paraformaldehyde (FD NeuroTechnologies, USA) and dyed with 0.05% crystal violet (Sigma-Aldrich, USA) at room temperature. Images were taken and the number of colonies with more than 50 cells was counted manually. Relative colony formation efficiency = (amount of colony in treated group/amount of colony in control group) × 100%.

### 3D formation assay

We simulated in vivo cancer cell survival when treated with valeric acid by performing a 3D formation assay. A hanging drop method has been successfully developed for generation of 3D spheroids that enables the growth of cells in a multidimensional and multicellular manner and facilitates cancer study and more efficient drug screening^37,38^. Briefly, MDA-MB-231 or MCF-7 cells were placed into each drop which contained 20 μl complete medium and 200 cells, respectively. 0.85 mM valeric acid or the same amount of ddH_2_O (control group) was then added into each drop. All drops were cultured in a humidified incubator at 37 °C with 5% CO_2_. Images were taken at different time points (24 h, 48 h, 72 h, 96 h). The cross-section area of 3D spherical formation was calculated by software ImageJ (version of 1.52a).

### HDAC colorimetric activity assay

The effect of valeric acid on histone deacetylase (HDAC) activity was assessed using the Colorimetric HDAC Activity Assay Kit (BioVison, Milpitas, CA, USA) according to the manufacturer’s instructions. Briefly, MCF-7 cells were treated with various concentrations of valeric acid for 48 h, at which point nuclear protein fractions were prepared using the Epiquik Nuclear Extraction Kit (Epigentek, USA) following the manufacturer’s protocols. 50 μg of nuclear extracts from treated cells were then diluted in 85 μL ddH_2_O. 10 μL of 10 × HDAC assay buffer was added followed by the addition of 5 μL of the colorimetric substrate. Samples were then incubated at 37 °C for 1 h. Subsequently, the reaction was stopped by adding 10 μL of lysine developer and left for an additional 30 min at 37 °C. OD values of samples were read by microplate spectrophotometer (Biotek, Winooski, USA) at the wavelength of 405 nm. HDAC activity was expressed as relative OD values per μg of protein sample. The assay was conducted in triplicate.

### Global DNA methylation assay

MCF-7 cells were treated with 5 mM valeric acid for 72 h and then collected for genomic DNA isolation using the QIAamp DNA Blood Mini Kit (Qiagen Sciences, Maryland, MD, USA). The Global DNA methylation levels were determined using the MethylFlash Global DNA Methylation Quantification Kit (Epigentek, NY, USA) according to the manufacturer’s instructions. The absorbance of the samples was measured on amicroplate reader at 450 nm. The experiment was performed in triplicate. For the OD intensity to be proportional to the amount of methylated DNA, the % 5-mC was calculated using the following formula: 5-mC% = [(sample OD-negative control OD) × P/(positive control OD-negative control OD) × S × 2] × 100%, where S represents the amount of input sample DNA (100 ng) and P represents the amount of input positive control DNA (5 ng).

### Genome-wide DNA methylation array

To further explore epigenetic impact of valeric acid on specific CpG sites in cancer related genes, we performed a genome-wide DNA methylation analysis. Genomic DNA of MCF-7 cells treated with 5 mM valeric acid for 72 h was isolated and purified using the QIAamp DNA Blood Mini Kit (Qiagen Sciences, Maryland, MD, USA) according to the manufacturer’s protocols. 50 ng of genomic DNA per sample was used for the analysis using the Infinium Human Methylation 450 BeadChips (Illumina, San Diego, CA USA). A methylation index (β) was used to estimate the methylation level of each CpG site using the ratio of intensities between methylated and unmethylated cytosines, which is a continuous variable between 0 and 1. 0 corresponds to a completely unmethylated site, while 1 corresponds to a completely methylated site. Significant methylation differences in methylation levels between treated and control group at each CpG site were obtained using The Illumina Custom Model.

### Network analysis

The Ingenuity Pathway Analysis software (IPA, Ingenuity Systems) (Redwood City, CA) was used to investigate whether differentially methylated genes were enriched for functional relationships between treated and control groups. Differentially methylated genes were input into the program and used to identify experimentally verified interactions using the Ingenuity Knowledge Base, a manually curated database of functional interactions extracted from peer reviewed publications^39^. *p* values for individual networks were obtained by comparing the likelihood of obtaining the same number of genes or greater in a random gene set as are actually present in the input set using a Fisher's exact test, based on the hypergeometric distribution.

### Statistical analysis

All statistics and figures were generated using the GraphPad Prism 7.00 software (www.graphpad.com) if not specified. All results were shown as mean ± standard error. All *p* values presented were two-sided and *p* < 0.05 was regarded as statistically significant.

### Ethical approval

This article does not contain animal studies and any studies with human participants performed by any of the authors.

## Supplementary Information

Supplementary Tables.
